# Identification of mitochondrial-related signature and molecular subtype for the prognosis of osteosarcoma

**DOI:** 10.18632/aging.205143

**Published:** 2023-11-16

**Authors:** Xiaokun Zhao, Jian Zhang, Jiahao Liu, Qi Chen, Changxiong Cai, Xinxin Miao, Tianlong Wu, Xigao Cheng

**Affiliations:** 1 Department of Orthopedics, The Second Affiliated Hospital of Nanchang University, Nanchang 330006, Jiangxi, P.R. China; 2 Jiangxi Key Laboratory of Intervertebral Disc Disease, Nanchang University, Nanchang 330006, Jiangxi, P.R. China; 3 Institute of Minimally Invasive Orthopedics, Nanchang University, Nanchang 330006, Jiangxi, P.R. China

**Keywords:** osteosarcoma, mitochondrial, prognostic signature, molecular subtype, biomarkers

## Abstract

Mitochondria play a vital role in osteosarcoma. Therefore, the purpose of this study was to investigate the potential role of mitochondrial-related genes (MRGs) in osteosarcoma. Based on 92 differentially expressed MRGs, osteosarcoma samples were divided into two subtypes using the nonnegative matrix factorization (NMF). Ultimately, a univariate, least absolute shrinkage and selection operator (LASSO), and multivariate Cox analysis were performed to construct a prognostic risk model. The single-sample gene set enrichment analysis assessed the immune infiltration characteristics of osteosarcoma patients. Finally, we identified an osteosarcoma biomarker, malonyl-CoA decarboxylase *(MLYCD),* which showed downregulation. Osteosarcoma cells proliferation, migration, and invasion were effectively inhibited by the overexpression of *MLYCD.* Our findings will help us to further understand the molecular mechanisms of osteosarcoma and contribute to the discovery of new diagnostic biomarkers and therapeutic targets.

## INTRODUCTION

Osteosarcoma is an aggressive malignancy of the skeletal system [1, 2]. It develops rapidly and has a poor prognosis and is associated with high mortality rates in children and adolescents [3, 4]. The prognosis of osteosarcoma patients has greatly improved over the last few decades, due to advances in chemotherapy and surgical resection [5, 6]. However, given the lack of early screening markers, it was estimated that about 20% of osteosarcoma patients have metastases at diagnosis, especially lung metastases [7]. There was also no significant improvement in the 5-year survival rate [8, 9]. As a result, identifying potential biomarkers for osteosarcoma is of great clinical importance.

Mitochondria have a vital role in the regulation of cellular life activities in eukaryotic cells [10, 11]. Mitochondria participate in the regulation of various physiological mechanisms such as the maintenance of Ca^2+^ homeostasis [12], cell death [13], and cell proliferation [14]. As a key site of cellular metabolism, major biochemical reactions such as the tricarboxylic acid cycle, oxidative phosphorylation, and fatty acid oxidation occur in mitochondria [15, 16]. In addition, despite the extremely active glycolytic function of tumor cells, many of them still have functional mitochondria, and their function is closely related to the migration and invasion of tumor cells [17, 18]. In osteosarcoma cells, it has been demonstrated recently that mitochondria can regulate various death mechanisms [19, 20]. Furthermore, through influencing metabolism, mitochondria can change how an osteosarcoma behaves biologically [21, 22]. However, the specific involvement of mitochondrial-related genes (MRGs) in the development and prognosis prediction of osteosarcoma remains unknown. Therefore, elucidation of the abnormal expression mechanism of MRGs and exploration of their specific role as potential biomarkers for the effective diagnosis and prognostic evaluation of patients with osteosarcoma are of utmost importance.

A systematic study was conducted on MRGs in osteosarcoma, identifying two subtypes of patients with different prognostic features. In this study, a prognostic model was established to distinguish patients with different risks of osteosarcoma. The model demonstrated robust prognostic performance and was validated in the GSE21257 cohort. Furthermore, levels of immune infiltration in patients with osteosarcoma were assessed and their relationship to risk was explored. Malonyl-CoA decarboxylase (*MLYCD*) was downregulated in osteosarcoma cells compared to osteoblasts and may serve as a promising biomarker. Last, overexpression of *MLYCD* inhibited proliferation, migration, and invasion of the osteosarcoma cell line MG63. In conclusion, this study offers novel evidence for the exploration of prognostic biomarkers and therapeutic targets for osteosarcoma.

## MATERIALS AND METHODS

### Data acquisition and preprocessing

**Figure 1:**
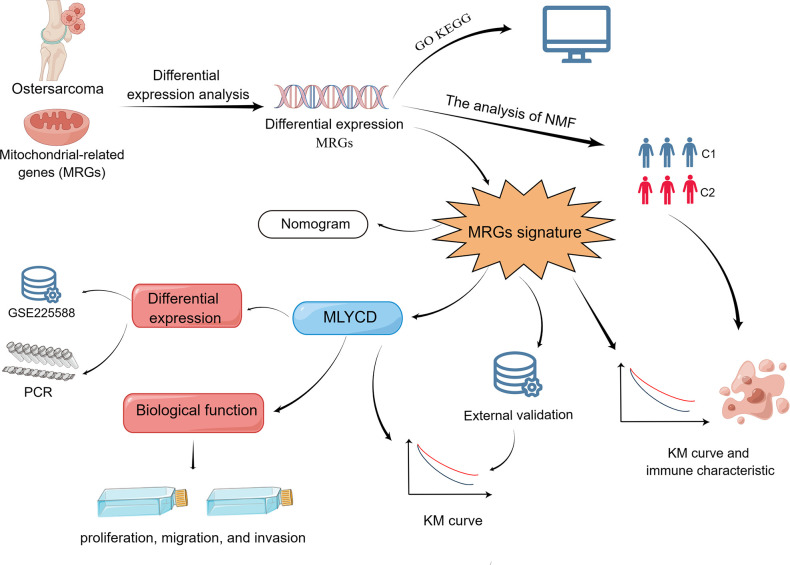
Flow chart of the program process.

The study’s summary is presented in Figure 1. From the TARGET database, osteosarcoma samples with clinical information and transcriptome data were obtained (https://ocg.cancer.gov/programs/target). Sequencing data and clinical information were complete in 84 cases. Using the GTEx database, we obtained gene expression data for musculoskeletal tissues (http://www.gtexportal.org), comprising a total of 396 healthy individuals. Prior to analysis, log-transformation of FPKM (fragments per kilobase of transcript per million mapped reads) transcriptome data and conversion to transcripts per million (TPM). A total of 1,136 mitochondrial-localized genes (Supplementary Table 1) were obtained from MitoCarta 3.0 (https://www.broadinstitute.org/mitocarta/), based on subcellular localization [23]. From the GEO database, we downloaded the GSE21257 dataset, which contained 53 osteosarcoma patients (https://www.ncbi.nlm.nih.gov/geo/). The GSE225588 dataset comprises six adjacent normal tissues and six osteosarcoma tissues, and GSE99671 dataset comprises eighteen adjacent normal tissues and eighteen osteosarcoma tissues.

### Biological functions and protein-protein interaction (PPI) networks

The GTEx gene expression dataset was merged with the TARGET gene expression dataset. “VennDiagram” package was used to identify MRGs from the three gene groups TARGET, GSE21257, and GTEX. Cut-off values for screening differentially expressed MRGs in osteosarcoma patients were set as expression >0.5, log2 FC>1, and adjusted P<0.05. Gene Ontology (GO) analysis was used to explore the biological function, pathway, or cellular localization of the enrichment. Kyoto Encyclopedia of Genes and Genomes (KEGG) was used to explore the enrichment pathways. Interactions between differentially expressed MRGs were analyzed by the STRING online tool (http://www.string-db.org/). Cytoscape software (version 3.7.2) was used for the construction and visualization of PPI networks.

### The clustering analysis

The subtypes based on MRGs in the osteosarcoma were determined using the non-negative matrix factorization (NMF) algorithm, through the “NMF” R package. The NMF algorithm is an effective method in bioinformatics to reduce the dimensionality of data such as gene expression microarrays. Clustering k-values ranging from 2 to 10 were tested, with the optimal k-value determined to be 2 based on the affinity coefficient. The levels of immune infiltration between clusters were compared using the MCPcounter algorithm.

### Development of the prognostic signature

A univariate Cox regression analysis was performed utilizing the “survival” R package, considering a significance level of p<0.05 to determine the prognostic relevance of 92 MRGs. The “glmnet” R package was employed to conduct 1000 iterations of LASSO analysis. Finally, through multivariate regression analysis, the key prognostic genes were determined and acquisition of risk coefficients for each gene were accomplished. Based on gene expression, a risk score equation was constructed as shown:


$$risk\;score=\sum_{i=1}^{n}\left(Coef_{i}*x_{i}\right)$$

The Coef*_i_* denotes the risk coefficients, and x*_i_* denotes the MRG expression value.

### Evaluation and validation of prognostic signature

Patients with osteosarcoma were subjected to risk score calculation, and subsequently sorted by ascending order. Based on the median, the patients were then categorized into low and high-risk groups. The analysis of prognosis disparity between the two groups was conducted, and time-related ROC curves were plotted using the “timeROC” R package. Univariate and multivariate Cox regression analyses were used to explore independent prognostic factors in clinical characteristics. By using the “rms” package, a nomogram was established, and calibration curves was used to compare actual and predicted results.

### Immune infiltration analysis

The relative levels of immune infiltration were estimated by utilizing the single-sample Gene Set Enrichment Analysis (ssGSEA). Enrichment scores derived from ssGSEA were then used to represent the relative abundance of each leukocyte subpopulation, which was normalized to a unit distribution ranging from 0 to 1. The biological similarity of infiltrating immune cells was estimated via the application of multidimensional scaling and a Gaussian fitting model.

### Cell culture

Two osteosarcoma cell lines (143B and MG63) and osteoblasts (hFOB 1.19) were obtained from the Cell Bank of the Chinese Academy of Sciences (Shanghai, China). Cells were cultured accordingly in DMEM (Procell, China) supplemented with 1% penicillin–streptomycin solution (Biosharp, China) and10% fetal bovine serum (FBS, Gibco, USA). A 37° C and 5% CO2 environment was used for osteosarcoma cells, and 34° C and 5% CO2 environment for osteoblasts. Trypsin-EDTA (Gibco, USA) was used to digest the cells.

### Extraction of RNA and RT-qPCR

Triazole treatment was applied to the cells, followed by extraction of total RNA and subsequent generation of cDNA through reverse transcription. Using qPCR kits and GAPDH as an internal reference, *MLYCD* expression was detected and quantified using relative qPCR. The primers used for *MLYCD* were those previously reported by Chen et al. [24]. The specific sequences are shown in Supplementary Table 2.

### Lentivirus infection

The ability of the stable transfection strain to elevate *MLYCD* levels was measured in the MG63 cell line and compared to a negative control lentivirus. The stable transfectants were selected with puromycin (1.0μg/mL), and the overexpression of *MLYCD* was confirmed by Western blot.

### Western blotting

Protein extraction from cells was performed using RIPA lysis buffer, followed by quantification using the BCA method. Subsequently, the protein samples were then separated by 10% SDS-PAGE gel electrophoresis and transferred onto a PVDF membrane. Blocking was performed with 5% skim milk, followed by incubation with anti-MLYCD (Proteintech: 15265-1-AP) antibody at 4° C overnight. To remove unbound antibodies, the membranes underwent three 10-min washes with TBST solution. Next, the second antibody was incubated for 2 h at room temperature. Finally, the expression of *MLYCD* was observed.

### CCK-8 and colony formation assays

A CCK-8 and colony formation assay were used to evaluate osteosarcoma cell proliferation. A total of 2000 cells with 100 μL medium were seeded in 96-well dishes. At the times indicated, each well was incubated for 2.5 hours at 37° C with 10 ul of CCK-8 solution. The absorbance of each well was measured at 450 nm. Regarding the clone formation assay, 1000 transfected cells were cultivated in 6-well culture dishes, and subsequent to a 14-day incubation period, the cells were fixed and stained.

### Cell migration and invasion assays

Transwell assays and wound healing assays were used to assess osteosarcoma cell migration and invasion. For the wound healing assay, approximately 95% confluence was reached after osteosarcoma cells were seeded onto a 6-well plate. The cell bed was scratch wounded with a 100-ul tip, after which the cells were cultured in serum-free medium. Intercellular spaces were observed and photographed with an inverted microscope within 0 and 24 h. For transwell experiments, osteosarcoma cells were seeded into the upper cell compartment, and 600 μL of medium containing 10% FBS was added to the lower cell compartment. The cells were fixed and stained 24 hours after incubation.

### Clinical specimens

Three samples of osteosarcoma and three samples of adjacent healthy tissues were collected.

### Immunohistochemical staining

The levels of *MLYCD* were assessed using immunohistochemistry in accordance with established protocols. The tissue sections of osteosarcoma underwent staining using a primary antibody, namely rabbit anti-*MLYCD* antibody (Proteintech, 15265-1-AP, diluted at a ratio of 1:200), after which the application of goat anti-rabbit IgG antibody.

### Statistical analysis

Data were analyzed using SPSS Statistics 22.0 and R software (v4.2.0). Students’ t-test was used to compare the differences between the two groups. A significance level of P<0.05 was used to determine whether the differences were statistically significant.

### Data availability

The datasets supporting the conclusions of this article are available in the TARGET-OS (https://ocg.cancer.gov/programs/target), the Gene Expression Omnibus (https://www.ncbi.nlm.nih.gov/geo/), the TCGA database (https://portal.gdc.cancer.gov/).

## RESULTS

### Identification and evaluation of subgroups

**Figure 2:**
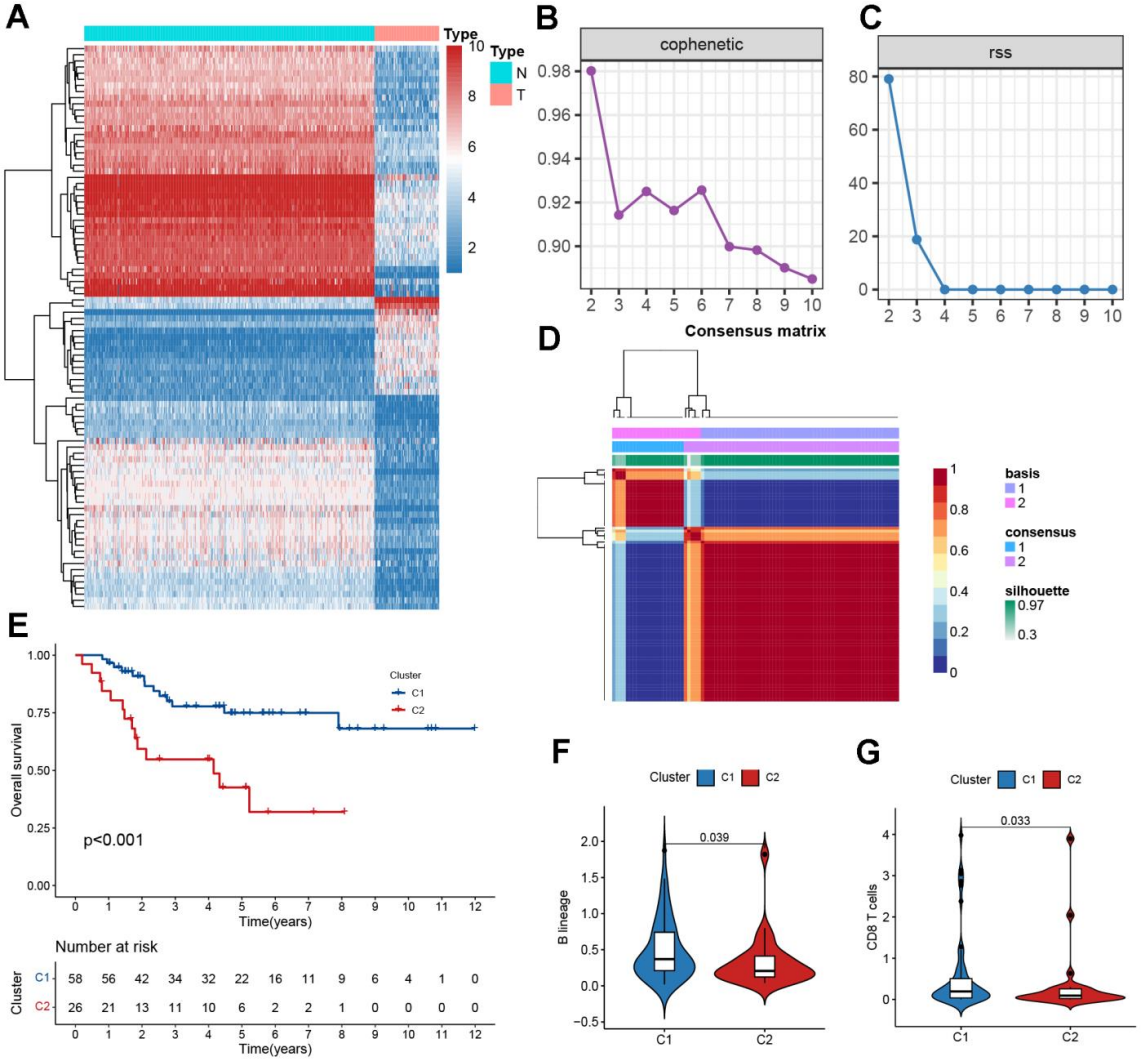
**Clustering based on NMF.** (**A**) The heatmaps of differentially expressed mitochondrial genes. (**B**) The cophenetic correlation coefficient is used to reflect the stability of the cluster obtained from NMF. (**C**) RSS is used to reflect the clustering performance of the model. (**D**) Consensus map clustered via the NMF algorithm. (**E**) Kaplan–Meier curve analysis for the two subtypes. (**F**, **G**) Immune scores of cells of the tumor microenvironment (TME) showing significant differences.

We considered the intersection of MRGs with the three datasets, and the Venn diagram showed that 804 MRGs were present in all three datasets (Supplementary Figure 1). After merging TARGET and GTEx and post-transforming them with TPM, a total of 111 MRGs were identified by differential analysis. To filter out low-expressed genes, we selected MRGs with expression greater than 0.5 in the osteosarcoma, and a total of 92 MRGs were selected and visualized using heatmaps (Figure 2A). The GO analysis showed 92 MRGs were mainly enriched in mitochondrial metabolic process (Supplementary Figure 2). The KEGG results showed that these MRGs were mainly enriched in oxidative phosphorylation (Supplementary Figure 3). Additionally, we conducted a PPI analysis; the larger the node, the darker the color, and the more interacting proteins. A significant role was played by *MLYCD* in the PPI network (Supplementary Figure 4).

To determine the molecular subtypes of patients with osteosarcoma, the following studies were performed. First, 92 differentially expressed MRGs matrices were constructed. Second, 84 samples from the TARGET cohort were included in the NMF analysis, and C1 and C2 were formed based on covariance and RSS when k=2 (Figure 2B–2D). We further evaluated the difference in survival between the two subtypes and found that the C1 subtype had a better survival rate than the C2 subtype (Figure 1E, P<0.001). The C2 subtype exhibited low levels of immune infiltration, with significant differences in B lineage and CD8 T cells, which was consistent with the concept of immune response inhibition in cancer (Figure 2F, 2G). In Sankey’s study, it was found that patients with the C1 subtype had a better prognosis (Supplementary Figure 5).

### Construction of prognostic signature

**Figure 3:**
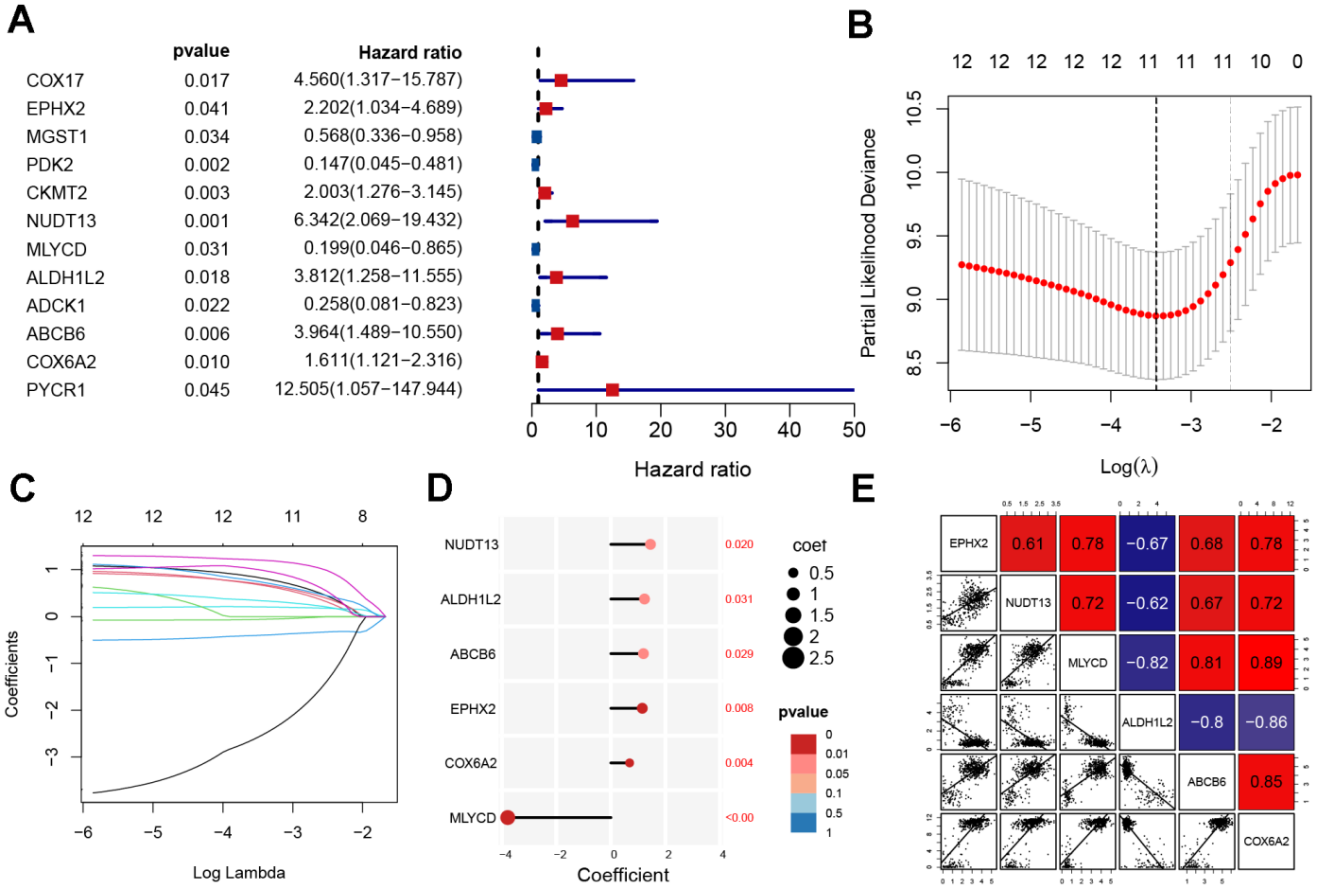
**Construction of mitochondrial-related gene signature.** (**A**) Univariate analysis of potential prognostic factors. (**B**, **C**) Lasso regression for MRGs in univariate Cox regression. (**D**) The coefficients and P-value of the six MRGs. (**E**) Correlation diagram of six gene expression levels.

For the purpose of determining prognostic risk models, 92 MRGs were analyzed. First, 12 prognosis-related MRGs were identified using univariate regression analysis (Figure 3A). Next, LASSO regression analysis was performed on the 12 MRGs to obtain 11 genes (Figure 3B, 3C). Finally, the modeling parameters and six model genes (*EPHX2, NUDT13, MLYCD, ALDH1L2, ABCB6, COX6A2*) were obtained using multivariate regression analysis. Upon visualizing the parameters of the model genes and their coefficients, it was observed that only *MLYCD* exhibited a negative correlation with the risk feature, whereas the other five genes exhibited a positive correlation with the risk feature (Figure 3D). We also performed correlation analysis and visualized the model genes (Figure 3E).

### Evaluation of the prognostic signature

**Figure 4:**
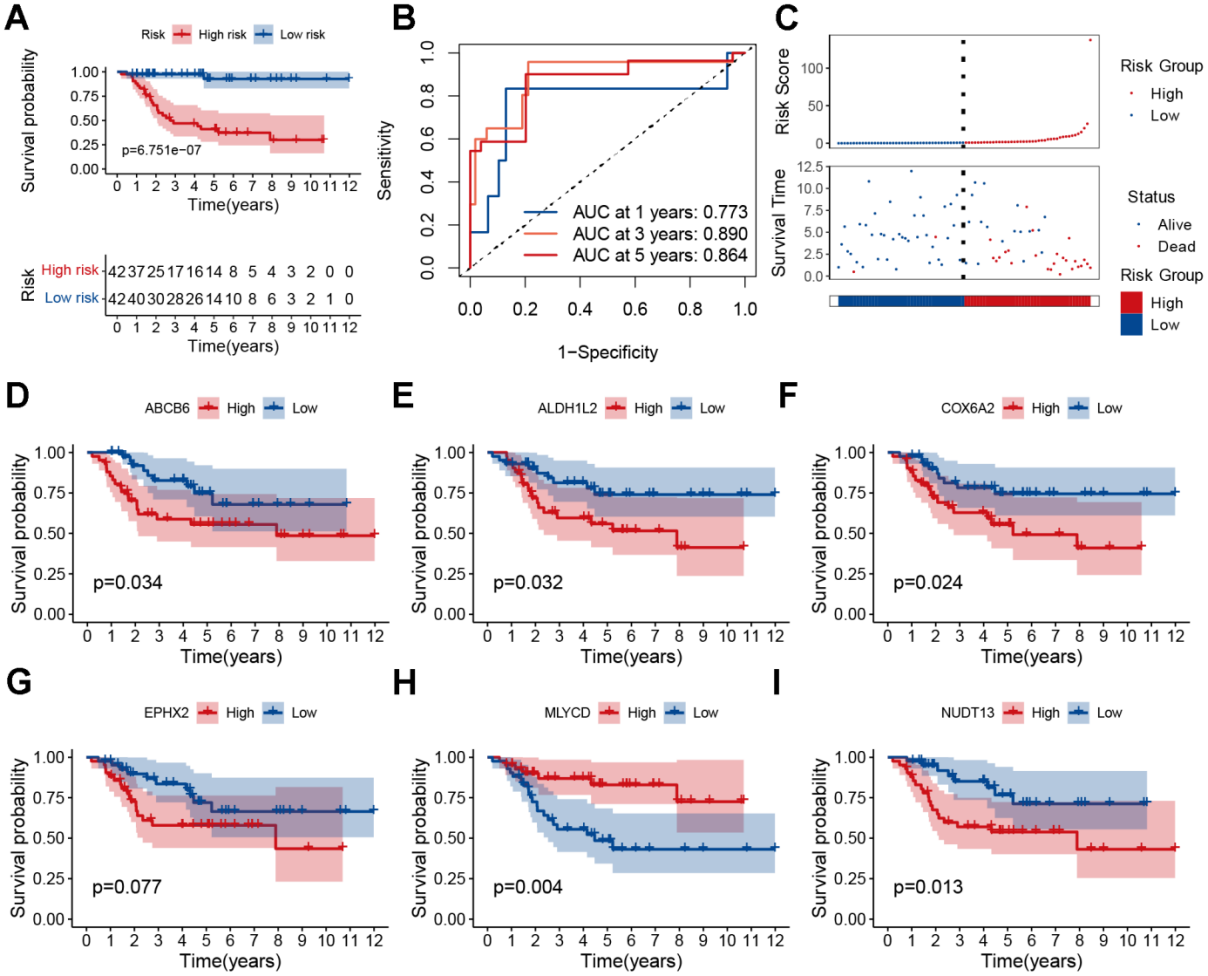
**Prognostic analysis of the MRGs signature.** (**A**) Kaplan–Meier survival curve analysis of patients in the high-risk group and low-risk group. (**B**) The AUC of time-dependent ROC curves. (**C**) The distributions of survival status and risk score. (**D**–**I**) Kaplan–Meier survival analysis of single genes in the TARGET cohort.

Osteosarcoma samples were divided into high-risk score and low-risk score groups according to the score medians, and survival curves were plotted using the Kaplan–Meier (KM) curve. The low-risk group had a significantly better prognosis than the high-risk group (Figure 4A, P<0.001). ROC analysis showed that the area under the curve (AUC) of the model was >0.77 at 1, 3, and 5 years (Figure 4B). Additionally, the distribution of risk scores and survival status revealed a higher risk score was associated with a higher probability of death. (Figure 4C). We performed principal component analysis (PCA) to demonstrate the predictive power of our model, which indicated significant differences in patient distribution (Supplementary Figure 6). We also performed survival analysis for each model gene and found that *NUDT13, MLYCD, ALDH1L2, ABCB6,* and *COX6A2* were associated with patient prognosis (Figure 4D–4I). In addition, *MLYCD* was shown to be the sole protective factor (P=0.004).

### Verification of the prognostic signature

**Figure 5:**
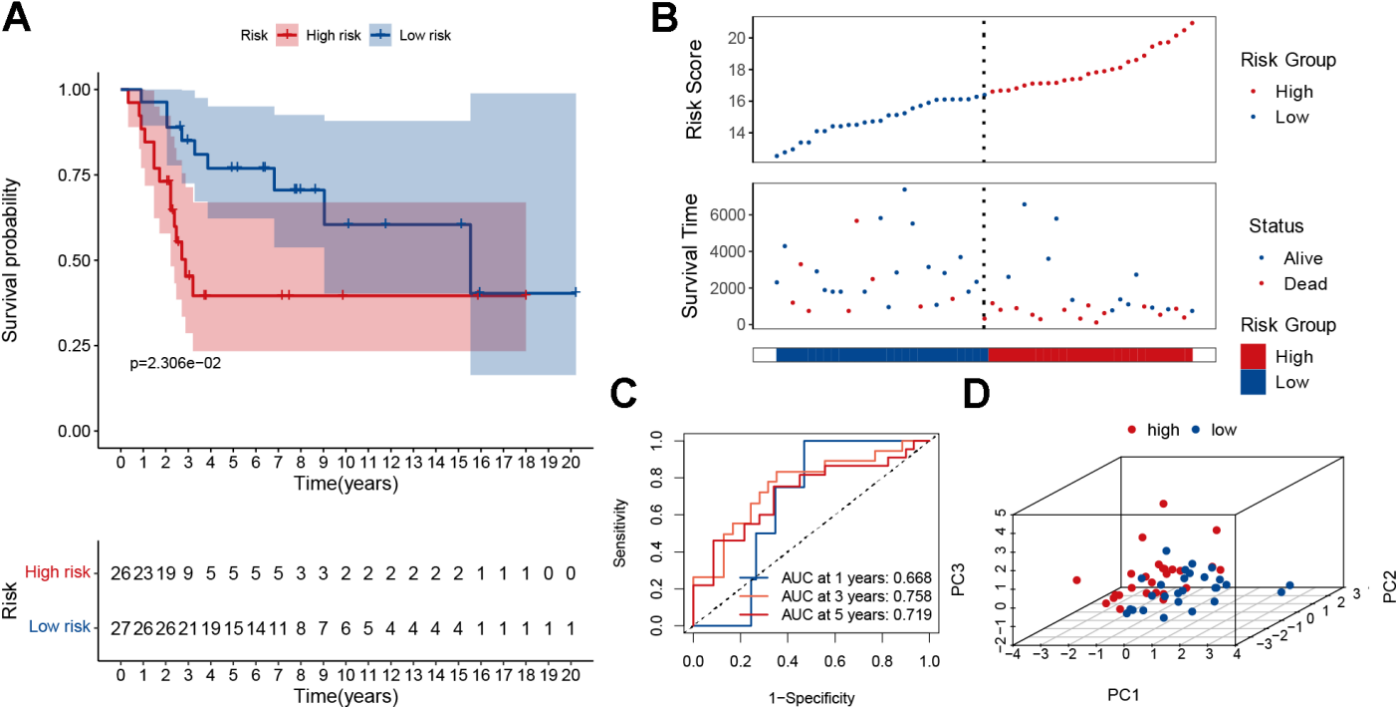
**Validation of the MRGs signature in the GSE21257 dataset.** (**A**) Kaplan–Meier survival curve analysis of patients in the high-risk group and low-risk group. (**B**) The distributions of survival status and risk score. (**C**) The AUC of time-dependent ROC curves. (**D**) PCA plot based on the MRGs signature.

To demonstrate the predictive power of our established model, the GSE2125 cohort was used as a test set for validation. It was also significant that the low-risk group had a better prognosis than the high-risk group (Figure 5A, p<0.05). As the patient’s risk increased, their mortality rate also increased (Figure 5B). The AUC for overall survival at 1, 3, and 5 years was greater than 0.66 (Figure 5C). PCA analysis showed that the model could well differentiate between high- and low-risk patients (Figure 5D). According to the single gene survival analysis, the GSE21257 cohort had similar results (Supplementary Figure 7). In summary, our model also had good predictive power in the external dataset GSE21257.

### Independent prognosis and clinical relevance

**Figure 6:**
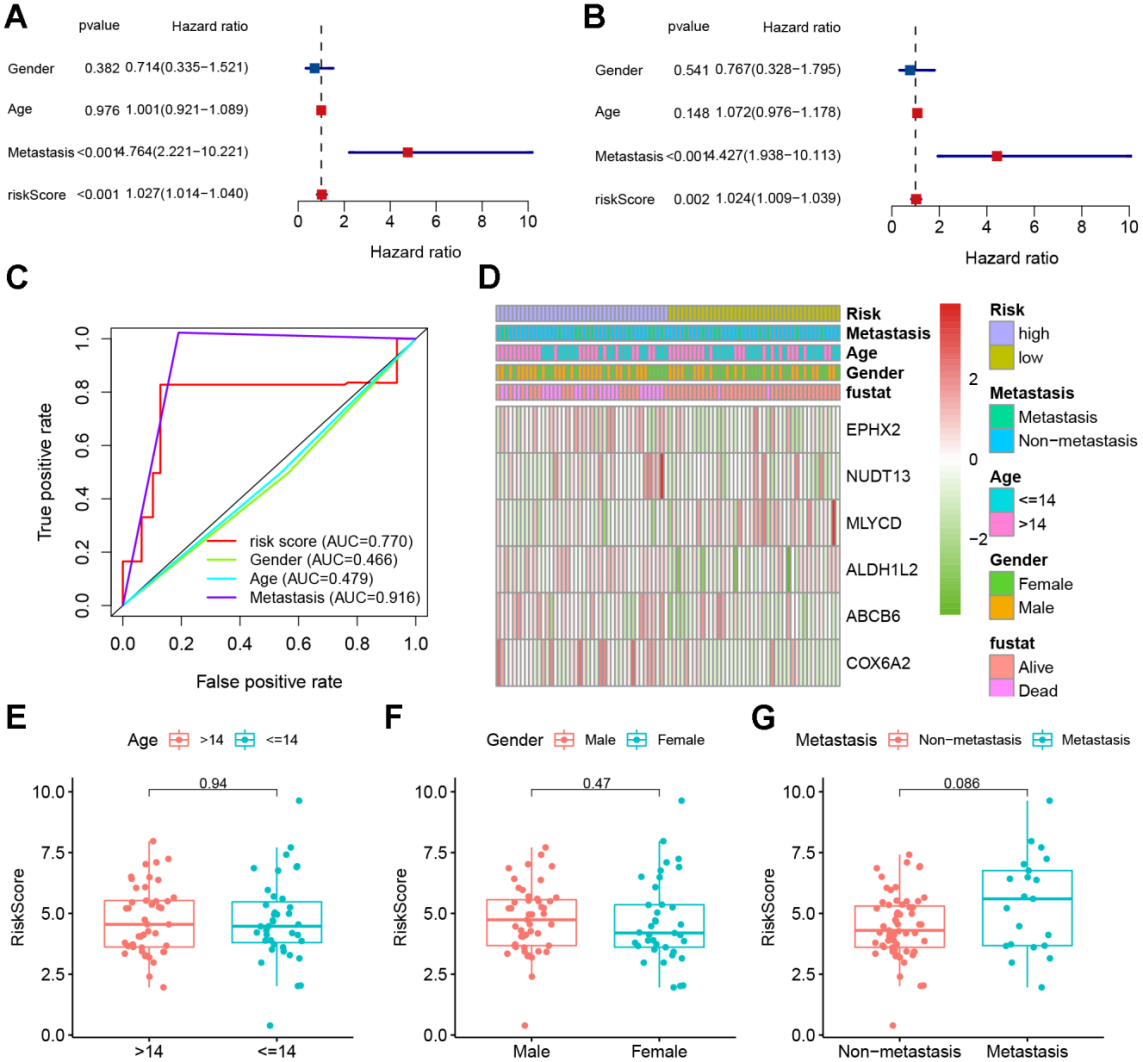
**Clinical correlation analyses.** (**A**) Univariate Cox analysis. (**B**) Multivariate Cox analysis. (**C**) AUC value predicts clinical characteristics and risk score. (**D**) Heatmap and the clinical characteristics of the two groups. (**E**–**G**) Relationship between risk score and clinical pathological factors.

Univariate Cox analyses were performed for the TARGET cohort after integrating the risk scores and clinical information. The risk score was a significant predictor of osteosarcoma, as shown in Figure 6A. Multivariate Cox analysis indicated that poor prognosis was significantly associated with risk score (Figure 6B). The AUC of the risk score was 0.770 (Figure 6C). This indicates that our risk model was capable of effectively predicting patient prognosis. After that, we plotted a heatmap of model genes versus clinical traits to roughly demonstrate the relationship between genes and clinical traits (Figure 6D). The *MLYCD* expression levels were negatively correlated with risk scores, suggesting that *MLYCD* may act as a protective factor; however, *ABCB6* was positively correlated with the risk score. Box plots showing the relationship between clinical and risk scores (including metastasis, sex, and age) revealed that age and sex did not differ between subgroups (Figure 6E–6J).

### Clinical nomogram and immune characteristics

**Figure 7:**
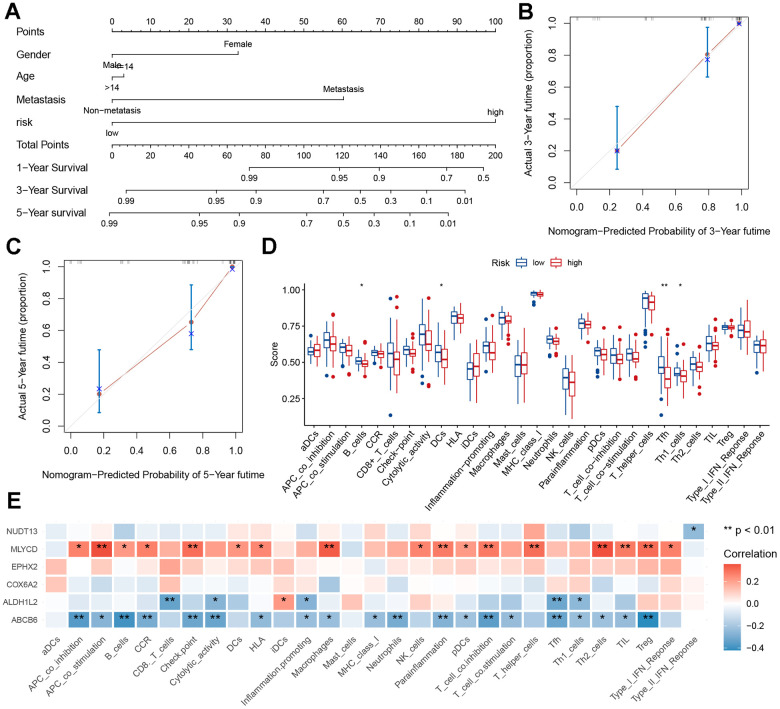
**Nomogram and immune signature of the risk model.** (**A**) Nomogram based on risk score, age, sex, and metastasis for predicting the 1-, 3-, and 5-year death rate. (**B**, **C**) Calibration plots of the nomogram for predicting the 3- and 5-year’ survival of osteosarcoma. (**D**) Relationship between risk score and immune cell infiltration and related functions via ssGSEA analysis. (**E**) Relationship between MRGs signature and immune cell infiltration and related functions via ssGSEA analysis.

To predict the risk of osteosarcoma in patients and understand the relationships between variables, a nomogram was constructed. The results showed that our constructed risk model contributed the most to predicting the outcome (Figure 7A). In addition, prediction accuracy of the columnar graph was evaluated using the C-index. The column line graph showed an ideal predictive ability for the 1-, 3-, and 5-years’ survival probability of patients (Figure 7B, 7C and Supplementary Figure 8). Using the TARGET dataset, an ssGSEA was conducted to evaluate immune cell infiltration levels. Immune cell infiltration and related functions in the high-risk group showed a downward trend (Figure 7D). To investigate the relationship between six genes (*EPHX2, NUDT13, MLYCD, ALDH1L2, ABCB6, COX6A2*) and immune infiltration, correlation plots were generated. Positive correlations were found between immune infiltration and *MLYCD* expression (Figure 7E). There was evidence to show that low-risk patients may experience higher levels of immune cell infiltration.

### *MLYCD* is lowly expressed in osteosarcoma

**Figure 8:**
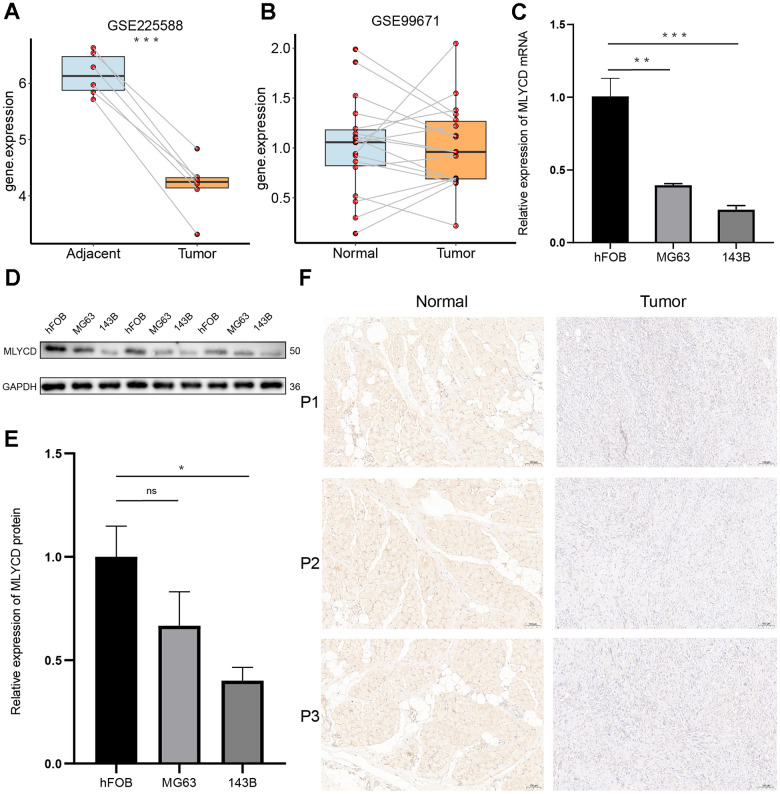
**The expression levels of *MLYCD*.** (**A**, **B**) The *MLYCD* expression level in osteosarcoma and adjacent paired samples, based on the GSE99671 and GSE225588 cohort. (**C**) The qRT-PCR result of *MLYCD* in hFOB 1.19, 143B, MG63 cell lines. (**D**, **E**) The western blotting result of *MLYCD* in hFOB 1.19, 143B, MG63 cell lines. (**F**) The expressions of *MLYCD* in tumor and adjacent normal tissues. *P<0.05, **P<0.01 and ***P<0.001.

Only *MLYCD* was identified as a protective gene (Figure 3D), so further experiments on this gene were performed. First, its expression was verified using the GEO dataset, and both GSE225588 and GSE99671 showed that *MLYCD* was lowly expressed in osteosarcoma tissues (Figure 8A, 8B). Next, the expression level of *MLYCD* gene in osteosarcoma cell lines (MG63 and 143B) and osteoblasts (hFOB 1.19) were verified by PCR (Figure 8C) and western blotting (Figure 8D, 8E). The expression level of *MLYCD* in osteosarcoma cells was lower than osteoblasts. Finally, by IHC staining of three pairs of osteosarcoma tissues and adjacent tissues, *MLYCD* was found to be lowly expressed in in osteosarcoma tissues (Figure 8F). All of the above results suggested that *MLYCD* was lowly expressed in osteosarcoma tissues.

### *MLYCD* suppressed osteosarcoma cell proliferation, migration, and invasion

**Figure 9:**
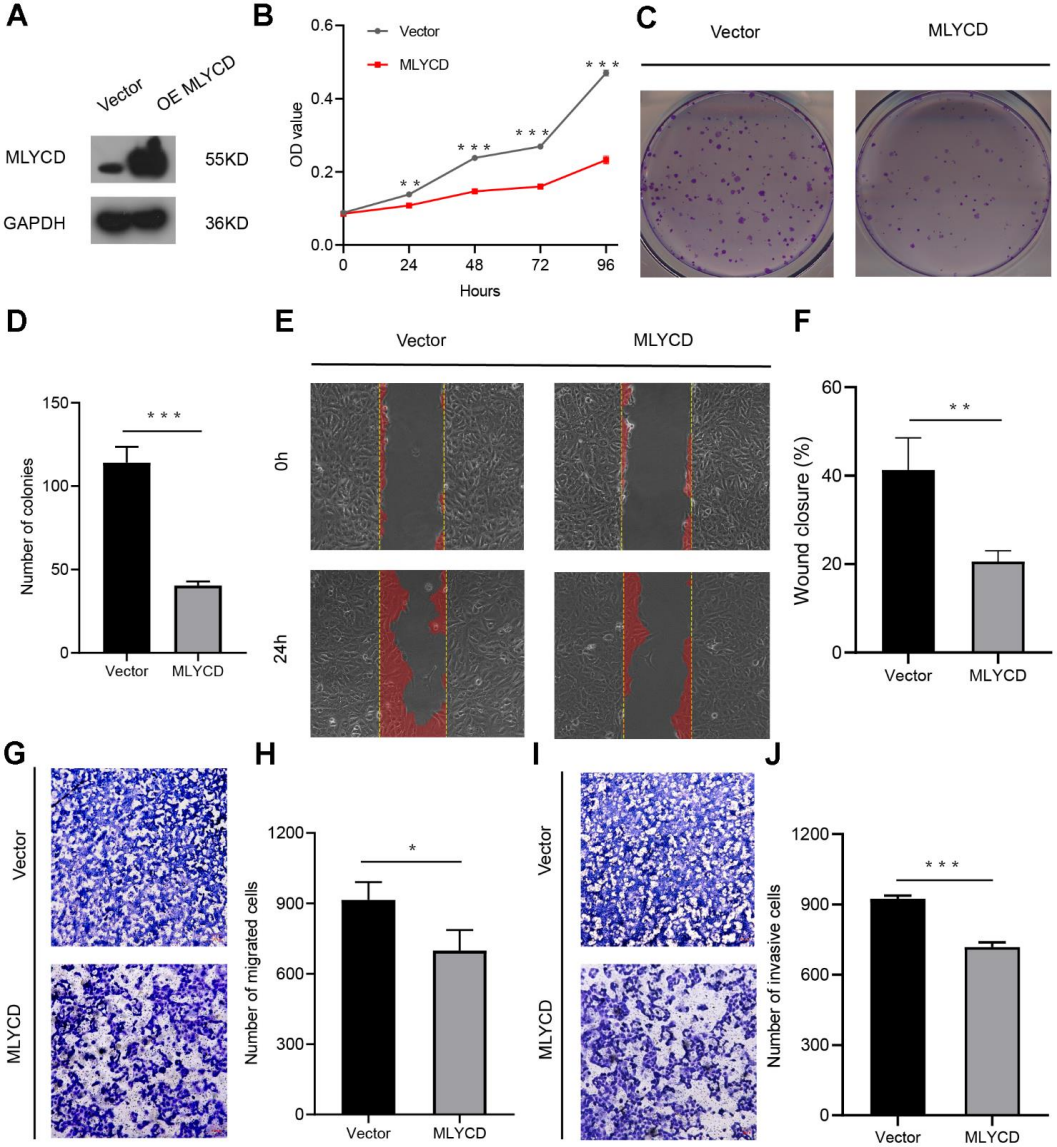
**The effect of *MLYCD* on osteosarcoma cell proliferation, migration, and invasion.** (**A**) Protein expression levels of *MLYCD* were measured by western blotting. (**B**–**D**) CCK-8 and colony-formation assays were used to assess osteosarcoma cell proliferation. (**E**, **F**) The wound healing assay was performed to estimate the effect of *MLYCD* overexpression on cell migration. (**G**–**J**) The transwell assay was conducted to assess the effect of *MLYCD* overexpression on osteosarcoma cell invasion and migration. Scale bar = 100um. *P<0.05, **P<0.01, and ***P<0.001.

To further investigate the role of *MLYCD* in osteosarcoma, overexpression of *MLYCD* in the MG63 cell line. Western blot confirmed the up-regulated expression of *MLYCD* in MG63 cells by lentiviral transduction (Figure 9A). CCK8 and plate colony formation assay showed that *MLYCD* overexpression led to decreased MG63 cell proliferation (Figure 9B–9D). In wound healing assays, *MLYCD* overexpression significantly suppressed MG63 cell migration (Figure 9E, 9F). In addition, transwell assays showed that *MLYCD* overexpression significantly reduced MG63 cell migration and invasion (Figure 9G–9J). In this study, the upregulation of *MLYCD* inhibits MG63 cell proliferation, migration, and invasion.

## DISCUSSION

Osteosarcoma is the most common primary malignant bone tumor worldwide, and it primarily affects children and young adults [25, 26]. Despite progress in surgical and comprehensive treatment strategies, prognostic markers and effective targeted therapies are still lacking [27–30]. Mitochondria are central nodes of cellular metabolism and fate determination [31]. Mitochondria are important bioenergetic organelles that produce ATP to meet the energy needs of cells. In addition, they are essential for the regulation of Ca^2+^ homeostasis and signaling [32], redox homeostasis [33], and for determining cell survival or death [34]. In osteosarcoma, synthesizing and catabolizing reactions provide cancer cells with the ability to cope with the demands of proliferation and adapt to unfavorable survival conditions. Therefore, identifying MRGs may help discover new therapeutic targets and improve patient survival.

In this study, we utilized the NMF algorithm to perform cohesive clustering on patients with osteosarcoma, identifying two distinct groups with different prognosis.

Subsequently, we constructed a robust prognostic model whose accurate predictive ability was validated using an external validation set. Additionally, we discovered that *MLYCD* serves as a protective factor in the model. The study demonstrated that MRGs can serve as independent predictors of osteosarcoma. A line chart was constructed using age, sex, signature, and metastasis to display good predictability of the 1-, 3-, and 5-year survival rates, which may help promote individualized treatment for osteosarcoma patients. Additionally, we found that high-risk groups exhibit a low degree of immune infiltration. Furthermore, we identified a potential therapeutic target, *MLYCD*, which is positively correlated with immune cells.

Various prognostic models associated with mitochondria have been developed. For example, Li et al. (2022) analyzed RNAseq data from 594 patients with Lung adenocarcinoma from TCGA and obtained a 6-gene signature with predictive prognostic value [35]. Jiang et al. (2021) also proposed a 16-gene prognostic marker as a potential survival predictive marker [36]. However, the results of these studies have not been validated in experiments. In addition, Zhang et al. (2022) established a 16-mitochondrial gene signature in osteosarcoma [37]. In this study, the TARGET tumor dataset and the normal tissue GTEx dataset were combined to develop a prognostic model for osteosarcoma osteosarcoma-related markers. The model was validated using an independent database and its reliability was verified in experiments.

*MLYCD* is a type of enzyme called propionyl-CoA carboxylase, which can catalyze the breakdown of propionyl-CoA into acetyl-CoA and carbon dioxide, providing a metabolic pathway for propionyl-CoA in mitochondria and peroxisomes. Mutations in the *MLYCD* gene can lead to a deficiency in propionyl-CoA carboxylase, a rare inherited metabolic disorder that presents as metabolic acidosis, hypoglycemia, and/or cardiomyopathy [38–40]. Currently, there is limited research on the role of *MLYCD* in cancers. Yizhak et al. found that silencing the *MLYCD* gene with small interfering RNA (siRNA) significantly inhibited the proliferation of RPMI-8226 and K562 [41], but *MLYCD* was not been studied in osteosarcoma at the time. Our KM survival curves showed that *MLYCD* was a good prognostic indicator and that its expression level was positively correlated with immune cells. Therefore, we investigated whether *MLYCD* was downregulated in osteosarcoma cells and found that overexpression of *MLYCD* significantly suppressed osteosarcoma cell proliferation, migration, and invasion. According to these findings, *MLYCD* may be a potentially useful therapeutic target.

The main limitation of our study was the small number of tumor samples. In future research, we plan to apply a larger sample size to build a more accurate prediction model. In addition, the mechanism of action of *MLYCD* on osteosarcoma progression needs further to be investigated by further molecular biology experiments.

## CONCLUSIONS

In summary, we identify molecular subtypes of osteosarcoma and establish prognostic models based on MRGs. The model showed good predictive performance in training and independent testing. We also confirmed the cancer suppressive function of *MLYCD* by proliferation and migration related experiments. The results of this study suggest that our signature may be used to evaluate the prognosis and provide a potential target for the treatment of osteosarcoma.

## Supplementary Material

Supplementary Figures

Supplementary Table 1

Supplementary Table 2
