# Supplementary material for: Identification of mitochondrial-related signature and molecular subtype for the prognosis of osteosarcoma
Source: Aging (Albany NY). 2023 Nov 16;15(22):12794–816. doi: 10.18632/aging.205143 (PMC10713410; doi:10.18632/aging.205143)
Supplement: Supplementary Figures [file aging-15-205143-s001.pdf]

SUPPLEMENTARY FIGURES

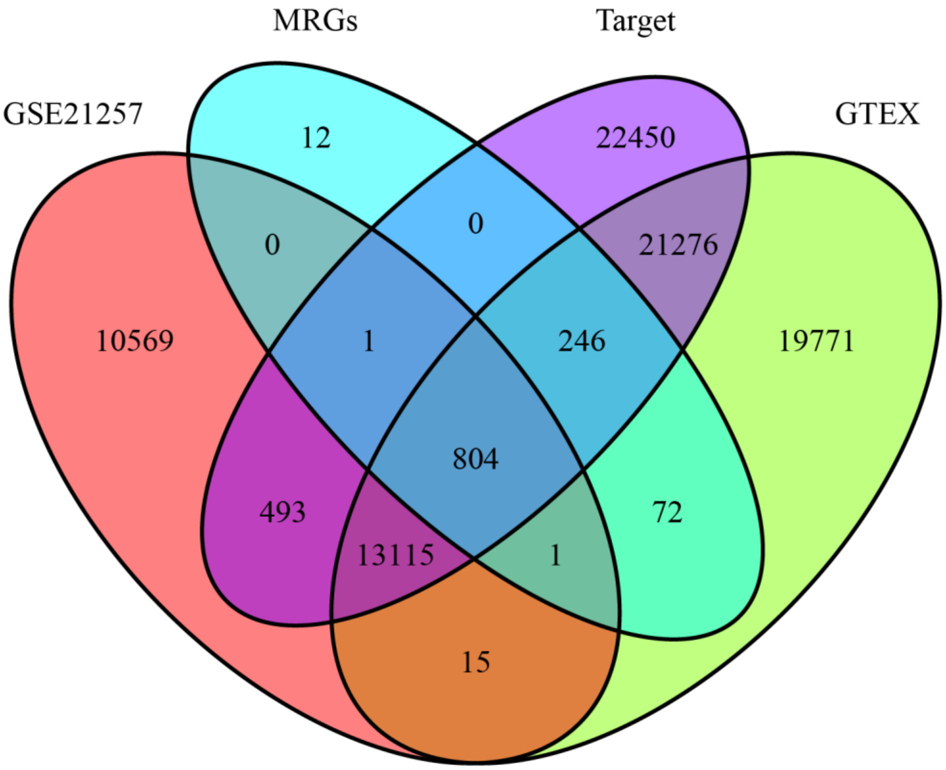

Supplementary Figure 1. Venn diagram of mitochondrial-related genes of the four data sets.

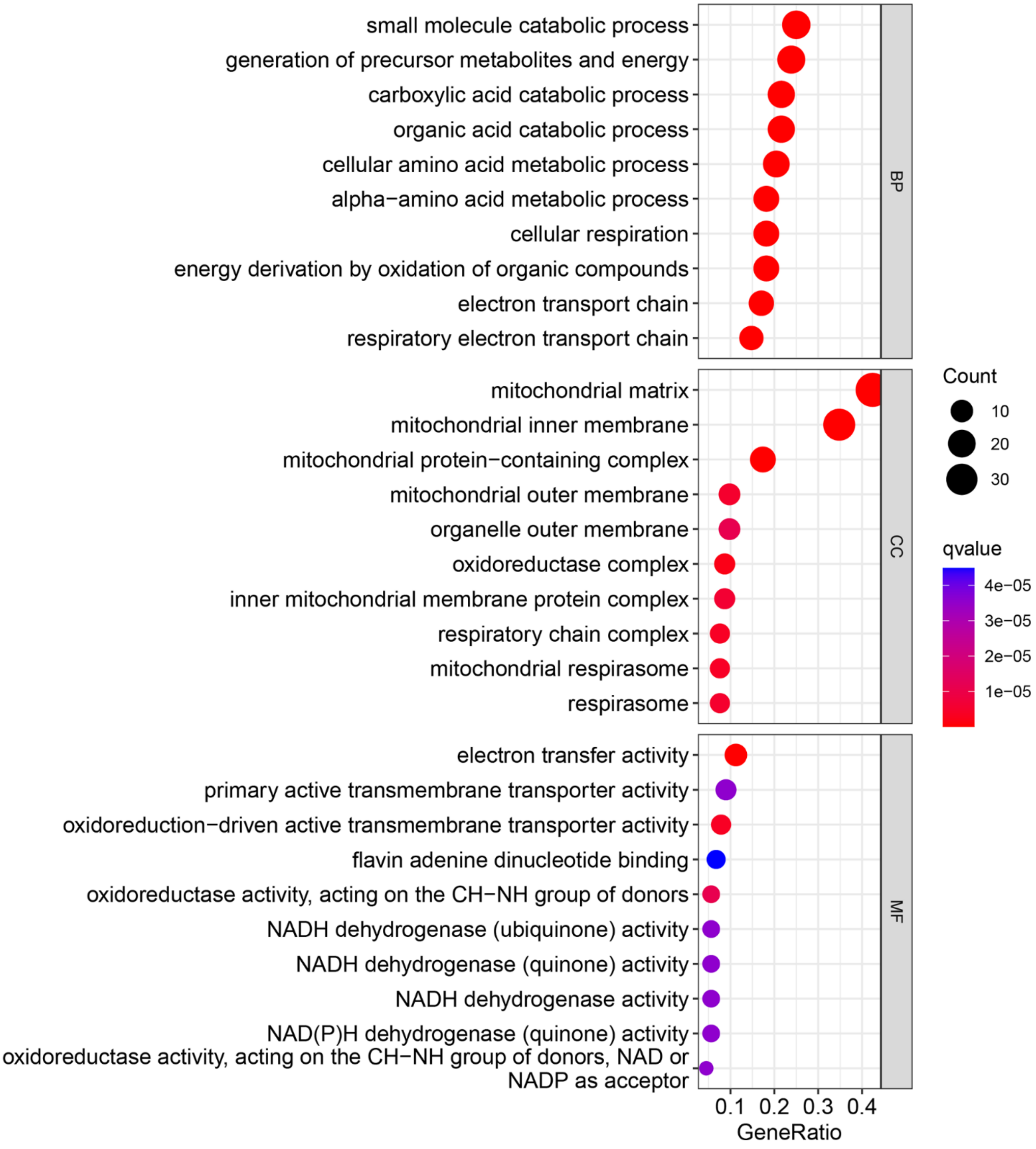

Supplementary Figure 2. Results of GO enrichment analysis.

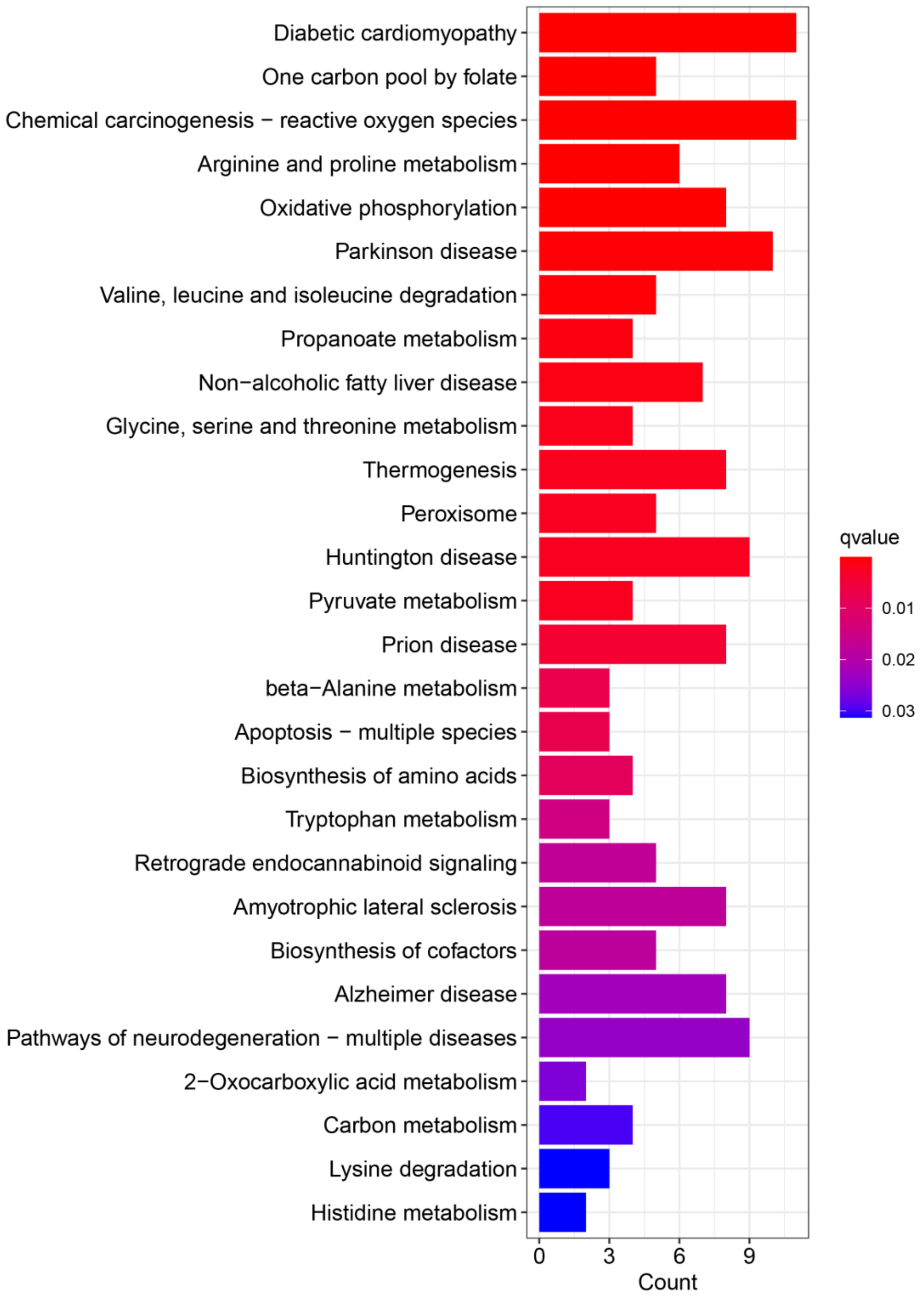

Supplementary Figure 3. Results of KEGG enrichment analysis.

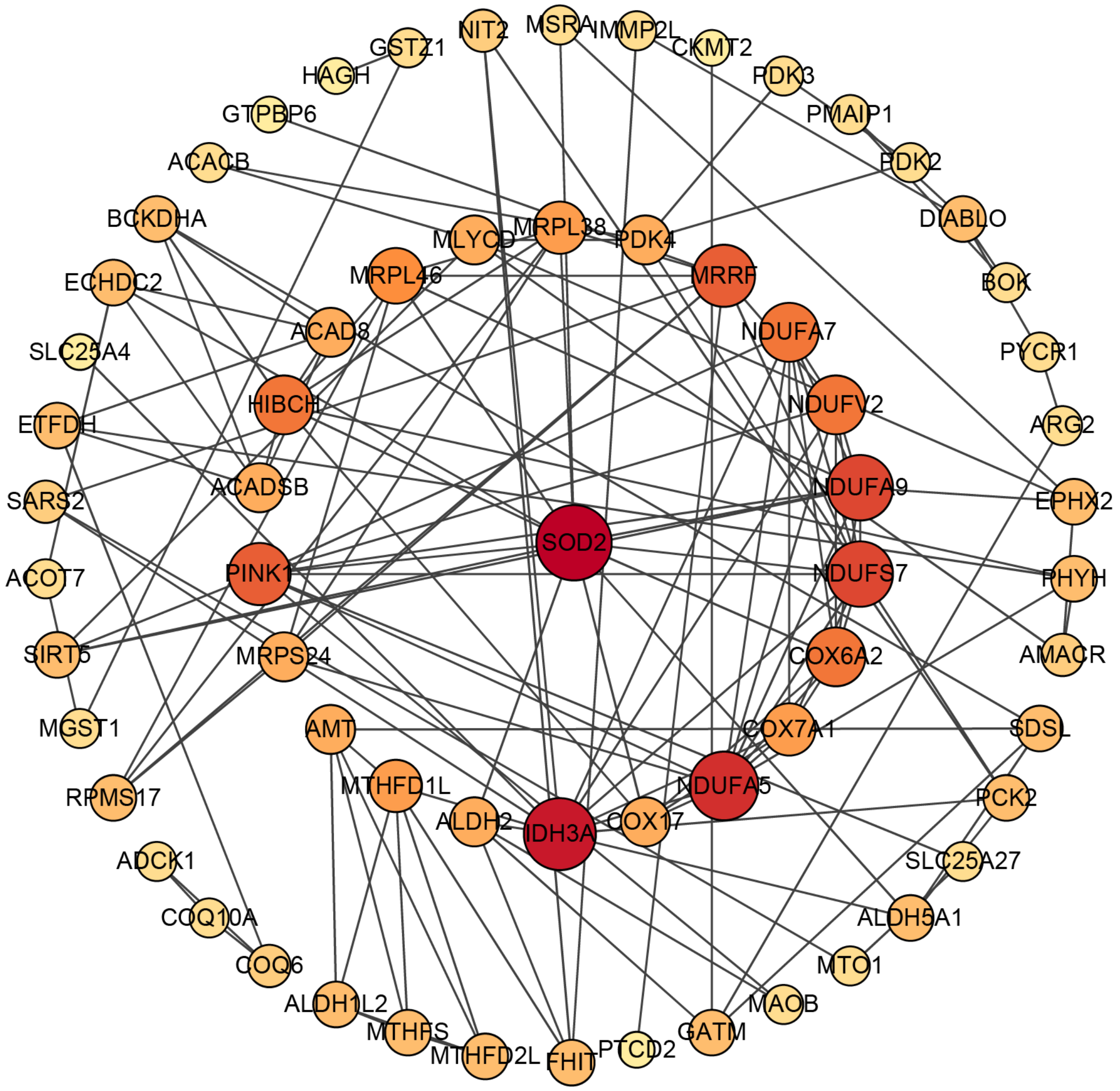

Supplementary Figure 4. PPI network of the 92 MRGs.

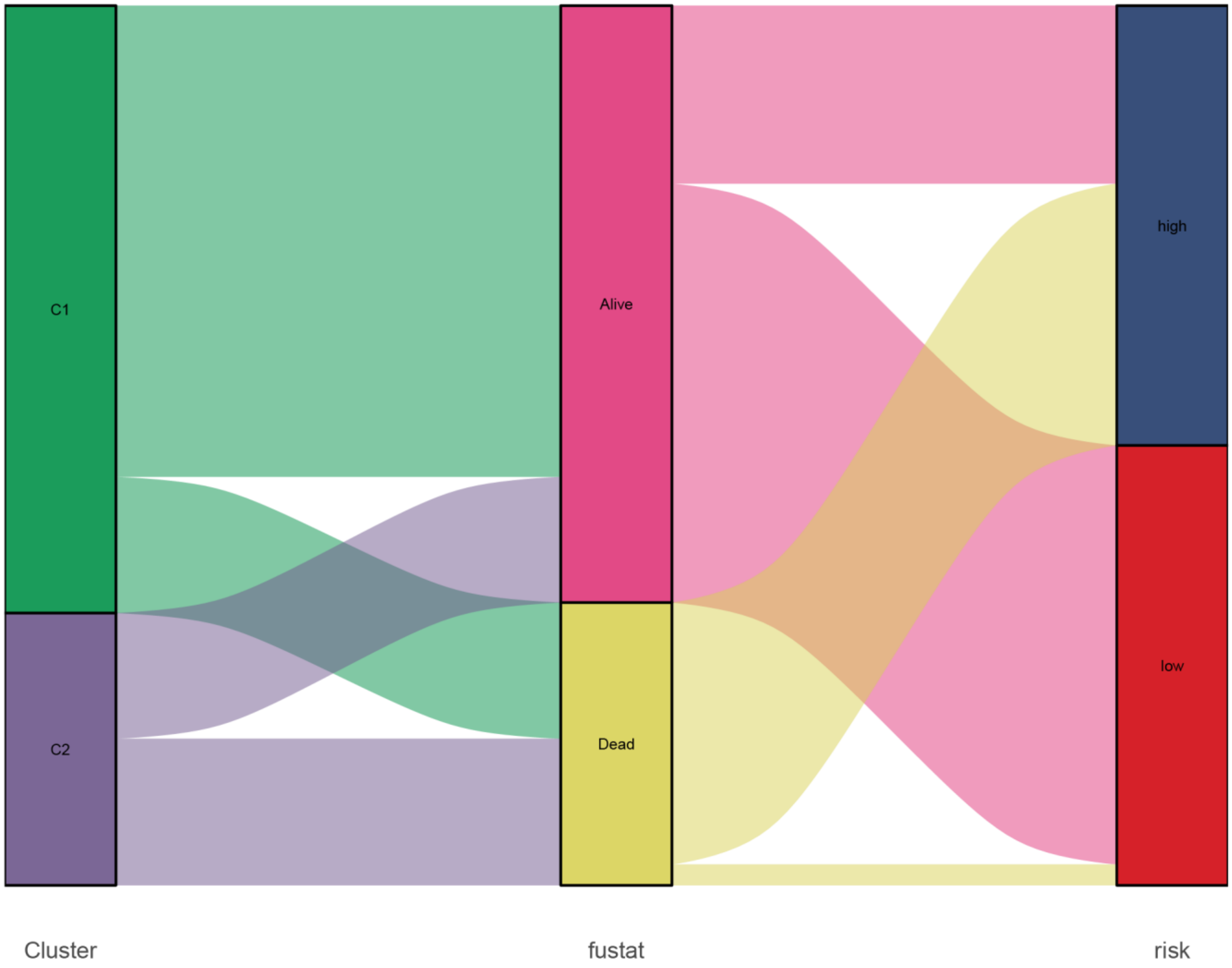

Supplementary Figure 5. Sankey diagram to reveal the distribution of samples.

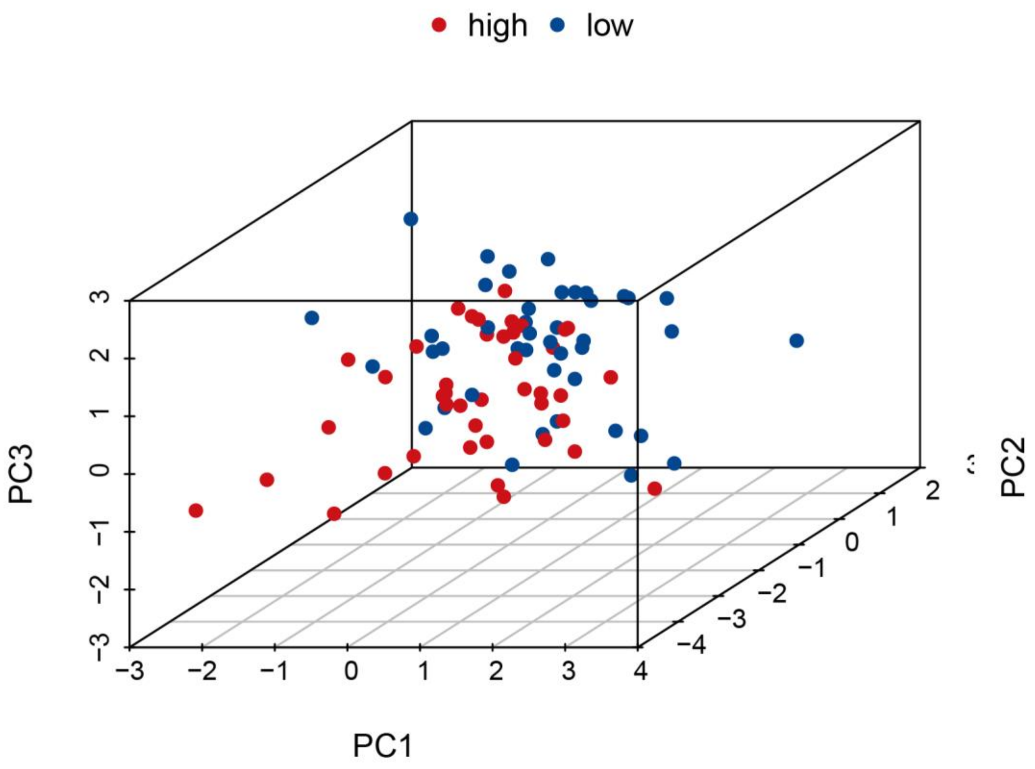

Supplementary Figure 6. Distribution of samples after PCA.

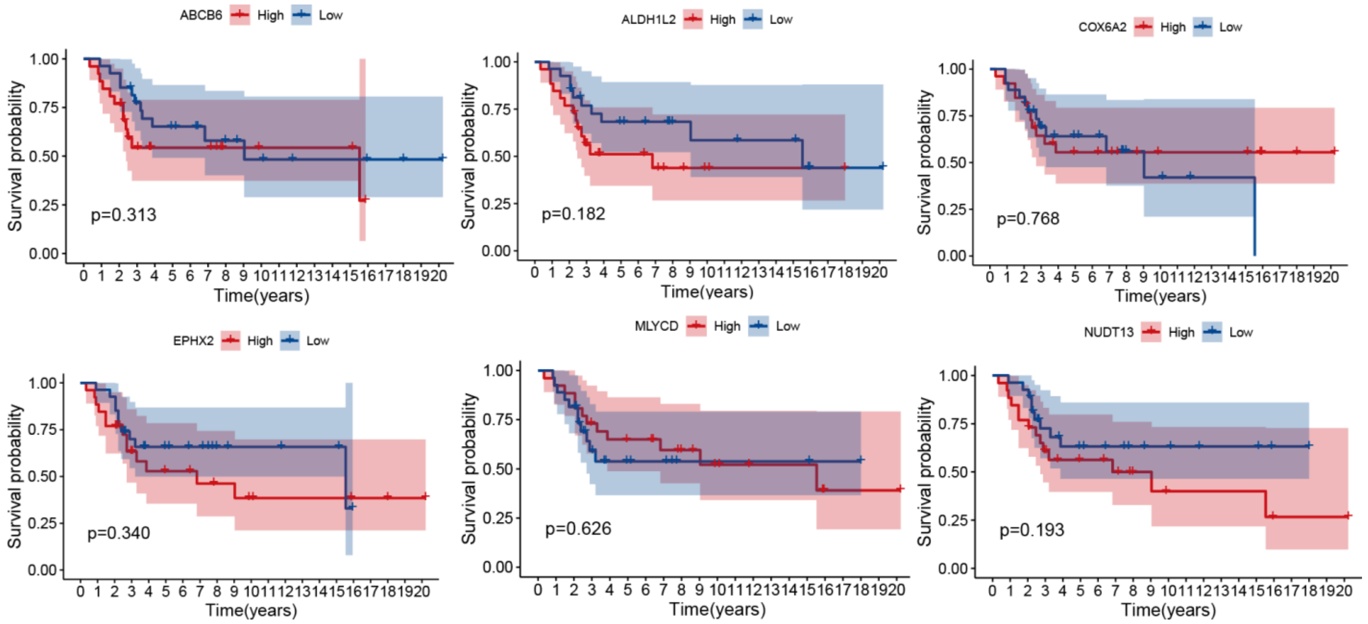

Supplementary Figure 7. Kaplan–Meier survival analysis of the six genes in the GSE21257 dataset.

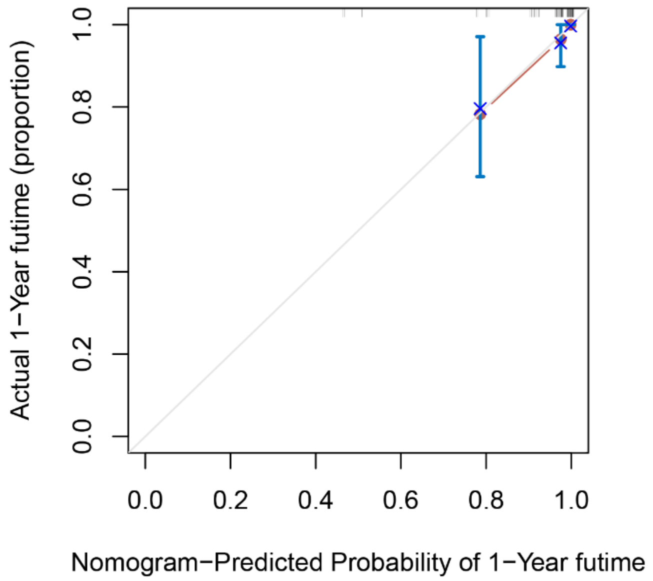

Supplementary Figure 8. The calibration curve at 1-year.
