# Supplementary material for: Identification of mitochondrial-related signature and molecular subtype for the prognosis of osteosarcoma
Source: Aging (Albany NY). 2023 Nov 16;15(22):12794–816. doi: 10.18632/aging.205143 (PMC10713410; doi:10.18632/aging.205143)
Supplement: Supplementary Table 2 [file aging-15-205143-s003.pdf]

Supplementary Table 2. The primer sequences of *MLYCD*.

Gene	Forward	Reverse
MLYCD	5- TTG CAC GTG GCA CTG ACT-3	5- GGA TGT TCC TTC ACG ATT GC-3
